# The Impact of 3D Printing on Mortar Strength and Flexibility: A Comparative Analysis of Conventional and Additive Manufacturing Techniques

**DOI:** 10.3390/ma19010212

**Published:** 2025-11-06

**Authors:** Tomas Gil-Lopez, Alireza Amirfiroozkoohi, Mercedes Valiente-Lopez, Amparo Verdu-Vazquez

**Affiliations:** Department of Building Technology, Technical School of Building Engineering, Polytechnic University of Madrid, Avenida Juan de Herrera 6, 28040 Madrid, Spain; alireza.amirfiroozkoohi@alumnos.upm.es (A.A.); mercedes.valiente@upm.es (M.V.-L.); amparo.verdu@upm.es (A.V.-V.)

**Keywords:** flexural strength, 3D printed mortar, traditional mortar, water-to-cement ratio, deformation, energy absorption

## Abstract

With the rise in additive manufacturing in construction, particularly 3D printing using extrusion-based mortars, there is an increasing need to optimize material properties. This study compares the mechanical performance of mortar specimens produced by traditional casting and 3D printing, with a focus on flexural behavior. A high-durability mortar with very low chloride and sulfate content, which produces less CO_2_ than standard Portland cement, was used. This study also explores the impact of varying water–cement (*w*/*c*) ratios to obtain a valid mix for both fabrication methods. The results show that the samples obtained by traditional processes and those produced through 3D printing exhibit distinctly different behaviors under bending stresses. In the case of the molded samples, the maximum stress ranged from 1.23 to 1.78 MPa, indicating good strength and uniformity within these materials. In contrast, the 3D-printed samples showed higher values but with greater variation, ranging between 2.77 and 3.76 MPa. This variation highlights the influence of the fabrication technique in 3D printing, which may contribute to either the superiority or limitations of these samples. In terms of deformation, molded specimens exhibited brittle failure with limited post-peak energy dissipation (0.11–0.22 kN.mm), whereas 3D-printed samples displayed a mixed brittle–ductile response and enhanced energy absorption (1.70–2.82 kN.mm). These findings suggest that traditionally obtained specimens are suitable for applications requiring predictable stiffness, while 3D-printed mortars are advantageous for applications demanding greater flexibility and energy absorption.

## 1. Introduction

Over the past decade, the construction industry has shown a growing interest in additive manufacturing (AM) techniques, particularly extrusion-based 3D-printable cementitious material (3DPCM), to address well-known challenges such as labor intensity, material waste, and the geometric limitations of traditional formworks [1,2,3,4,5,6,7,8,9]. This contrasts with conventional cast mortars, where molds or formworks are prepared and then filled.

3DPCM is produced using a layer-by-layer deposition process driven by computer-aided design (CAD). The development of Building Information Modeling (BIM) has facilitated better information management [10,11,12]. The use of this technology has important advantages for the construction sector, such as a reduction in labor, a reduction in execution times, a reduction in carbon dioxide emissions, and an improvement in thermal properties, among others [13,14,15,16,17]. Additionally, this approach enables the creation of complex geometries that would be otherwise impractical or prohibitively expensive using traditional methods [18,19,20,21].

Despite the advantages of this form of manufacturing, widespread adoption of 3DPCM has been held back by issues related to material consistency, printability and mechanical properties [22,23,24]. Unlike traditionally obtained mortars, which have been tested and refined over decades of industrial practice, printable cements demand a careful balance of fresh-state rheology (flowability, viscosity, open time) and hardened performance (compressive, flexural, and tensile strengths) [25,26,27]. The microstructural arrangement and packing density of granular constituents play a crucial role in governing these rheological properties, as particle packing affects both the deformation behavior and the mechanical stability of the material under load [28,29]. Research is currently underway to improve the efficiency of 3D printed structures to ensure accurate and efficient mortar deposition [30]. This delicate balance becomes even more critical when the water-to-cement (*w*/*c*) ratio varies, significantly influencing both pumpability and final strength [31,32].

The water-to-cement ratio is vital in conventional concrete and mortar mixtures, influencing workability, hydration kinetics, and mechanical outcomes [33,34,35,36,37,38,39,40]. In 3DPCM, however, the effect of the water/cement ratio is, if possible, of even greater importance [41,42,43], because in addition to the above, it requires competing requirements of:

(a) Extrudability: The mortar must be fluid enough to be pumped through a nozzle [44,45,46].
(b) Buildability: Each layer must support the weight of subsequent layers without excessive deformation [47,48,49,50].
(c) Bond Strength: A suitable consistency is needed so newly deposited layers adhere properly to the previous layers [51,52].

A high *w*/*c* ratio can improve flow but may weaken the final matrix and lead to bleeding or segregation [53,54,55,56]. In contrast, a low ratio can yield a stiff mixture that is difficult to extrude and prone to “cold joints” at layer interfaces [57]. Optimizing *w*/*c* is central to achieving consistent mechanical performance of printed large-scale elements [58,59,60,61].

In order to construct both large buildings and civil works, there is an increasing need, first, to ensure reliable mechanical properties and, second, to optimize both economic and environmental costs [62,63,64].

While compressive strength has long been the standard metric for evaluating the load-bearing capacity of mortar, flexural strength (or modulus of rupture) provides a more direct indicator of how mortar behaves under bending stresses. In many architectural and structural applications—beams, slabs, and thin-walled elements—tensile and bending stresses become critical design considerations [65,66,67]. For 3D-printed elements, which rely on freshly deposited layers bonding together, flexural behavior is also sensitive to layer interfaces and print path orientations [14,68,69]. Minor inconsistencies in the layer bonding process can create planes of weakness, potentially reducing the overall integrity of the structure [17,70,71,72].

Among the parameters that influence the bond strength between layers of different phases are printing speed, print layer thickness, nozzle height and curing conditions [71,73,74,75,76]. Another determining factor is the reduction in flexural strength caused by the increased porosity at the interfaces of the printed mortar layers [77]. Additionally, research to date reveals that flexural rigidity varies with printing direction, due to the anisotropy of the printed material [23,78,79,80,81]. This anisotropy not only influences the strength of the material, but also the durability of the material [82,83]. However, it is often difficult to vary the printing direction of the material when printing large-scale buildings, since it is usual to print the load-bearing elements of a building from bottom to top by adding horizontal layers.

In an attempt to solve this problem, additives [84,85,86] or recycled aggregates [87,88,89,90,91,92] are used to improve the mechanical characteristics of mortars by increasing their strength [93,94,95], reducing their weight [96,97,98], or enhancing their fire resistance [99]. There are also studies on reinforcement methods compatible with 3DPCM to increase the flexural strength of the material. The development of BIM technology has enabled improved automation in the integration of metallic reinforcement bars [100].

Furthermore, the optimization of printable mortars critically depends on the binder’s chemical composition. Specifically, fine-tuning oxide ratios such as CaO/Al_2_O_3_ and SiO_2_/Al_2_O_3_ in slag- and fly ash-based systems has been shown to regulate reactivity, phase formation, and microstructural development, thereby enhancing workability, controlling deformation, and mitigating cracking under shrinkage or post-peak stress conditions. Moreover, practical 3D printing applications often require functional upscaling through in-process reinforcement; novel automated bar insertion methods have demonstrated notable improvements in the structural performance of complex elements, including reinforced concrete beams [101].

However, in addition to the above, there are possibilities to improve the parameters involved in the printing process of the material (interlayer interval time, printing speed, hydration of the material, …), with the aim of making it similar to mortar obtained by a traditional process. Consequently, improving or maintaining flexural strength in 3D-printed mortar is a key step in ensuring reliable structural performance.

Motivated by the need to balance printability with structural performance, this research compares the flexural strength of a cement-based mortar produced traditionally and by 3D printing, under controlled laboratory conditions. Specifically, the study aims to:

(a) Evaluate how varying *w*/*c* ratios affect both printability and flexural behavior of the mortar, and
(b) Compare the mechanical performance (particularly flexural strength) of 3D-printed mortar samples to that of cast specimens under standardized testing protocols.

By focusing on the effect of *w*/*c* ratio on overall printability and mechanical performance, this study aims to provide clear guidelines for selecting or adjusting mix designs in large-scale 3DPCM applications.

## 2. Materials and Methods

This section presents the materials used (including mortar composition and preparation), the standards applied, flexural strength test methods, and other relevant properties compared between traditionally molded and 3D-printed specimens.

### 2.1. Mortar Composition

It is intended to select a proprietary bagged dry mortar to ensure a consistent composition of cement and sand in all specimens.

The mortar used must be suitable for both traditional preparation methods and processing in 3D printers.

The goal is for the chosen material to have multi-purpose use, i.e., it must be suitable for both structural and non-structural building components, appropriate for both interior and exterior applications.

Considering the aforementioned criteria and existing studies on this subject, the mortar used must meet the following requirements:

- It should be a high-performance, multi-purpose material, capable of being used for architectural, structural, industrial, and design purposes.
- It should be durable in all environments; therefore, it must be free from metallic compounds, with very low chloride and sulfate content.
- It should be capable of producing highly durable objects with low shrinkage.
- It must set within 3–5 min and acquire sufficient strength within one hour.
- It must have the ability to 3D print at speeds between 300 and 600 mm/s.
- It must be capable of producing layers between 30 and 50 mm in height.
- It should withstand the weight of a printed layer within a 10-s interval.
- It should help reduce the carbon footprint and minimize environmental impact by emitting less CO_2_ during manufacturing than other cements.

Among the available mortars, CyBe’s bagged dry mortar (CyBe Construction B.V., Oss, North Brabant, Netherlands) has been selected for this research because it meets all the aforementioned requirements. It is marketed in 25 kg (55 lb) bags [102]. Furthermore, according to its specifications, the shelf life before adding water is 6 months, provided it is stored in a cool, dry, and humid place away from direct sunlight.

The material properties are sourced from the mortar’s data sheet (Table 1):

The data obtained through laboratory analysis show a Loss on Ignition (LOI) value of 4.38%. The Blaine fineness was measured at 5200 cm^2^/g. The sand used had a particle size ranging from 0.15 to 2 mm. The oxide composition determined by X-ray fluorescence (XRF) is presented in Table 2:

Although there are other mortars available on the market, this one has more favorable parameters for this study. This proprietary blend of cement and sand is specifically designed for fast-setting, high-strength applications in extrusion-based 3DPCM and is also suitable for mortar production via traditional methods. Furthermore, according to the technical data:

- The mortar sets in approximately 3 min and reaches structural strength within 1 h, enabling rapid layer deposition. This parameter is crucial for the 3D printing of the material used in this research.
- It is formulated for layer heights up to 50 mm and printing speeds up to 600 mm/s. After conducting several tests, a layer height of 30 mm and a printing speed of 400 mm/s were chosen for this research, as these settings provided the most favorable buildability.
- A recommended interlayer interval of about 10 s is suggested to promote proper bonding. This parameter is feasible for this research to ensure adequate bond strength.
- The aggregate particle size is controlled (0–3 mm) to ensure pumpability and prevent nozzle clogging (extrudability).
- This mortar offers high durability with very low chloride and sulfate content, producing less CO_2_ than standard Portland cement. This characteristic was specifically sought for this research to reduce the ecological footprint by decreasing CO_2_ emissions, aligning with previous work [116,117].

### 2.2. Water-to-Cement Ratio

Prior to the fabrication of the specimens, both traditionally and through 3D printing, a specific water-to-cement (*w*/*c*) ratio must be determined that is valid for both fabrication methods.

In this case, the *w*/*c* ratio for the 3D-printed specimens is more restrictive, as it is a critical parameter influencing both printability and the mechanical performance of the hardened mortar. In this study, trial batches were prepared with *w*/*c* ratios ranging from 0.13 to 0.25, based on the following considerations [52,70]:

(a) Extrudability: The mortar must remain sufficiently fluid to be pumped through a low-pressure system without clogging.
(b) Layer Stability: Each deposited layer needs to support subsequent layers without excessive slumping.
(c) Interlayer Bonding: A proper moisture level ensures that freshly deposited layers can fuse effectively with the underlying layers.
(d) Mechanical Strength: Excess water can weaken the hardened matrix, while too little water may impede proper hydration and workability.

As the *w*/*c* ratio influences the mechanical properties of the mortar, this value should be the same for both traditionally manufactured and 3D-printed specimens. In this study, specimens were tested with *w*/*c* ratios of 0.13, 0.15, 0.18, 0.20, and 0.25.

**Figure 1:**
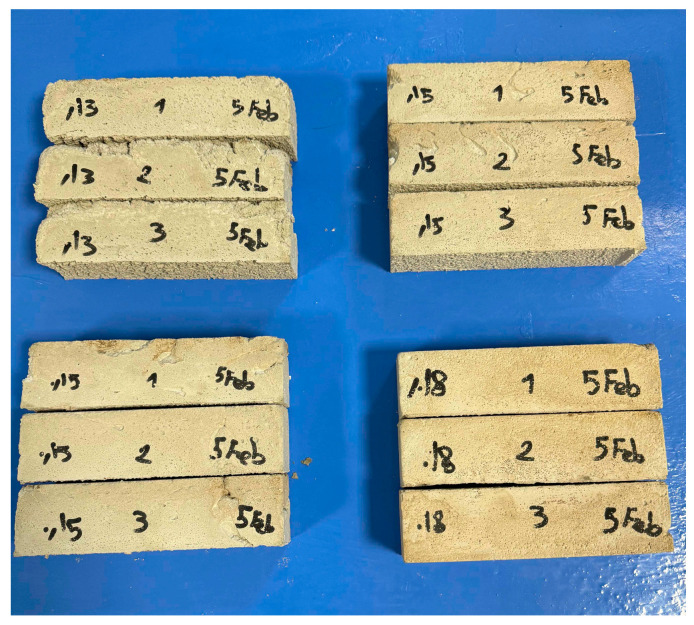
Mortar specimens with identical dimensions (20 cm × 5 cm × 5 cm).

Before manufacturing the final specimens, mortar samples of identical dimensions (20 cm × 5 cm × 5 cm) were prepared and tested with the five water contents mentioned above (Figure 1).

**Figure 2:**
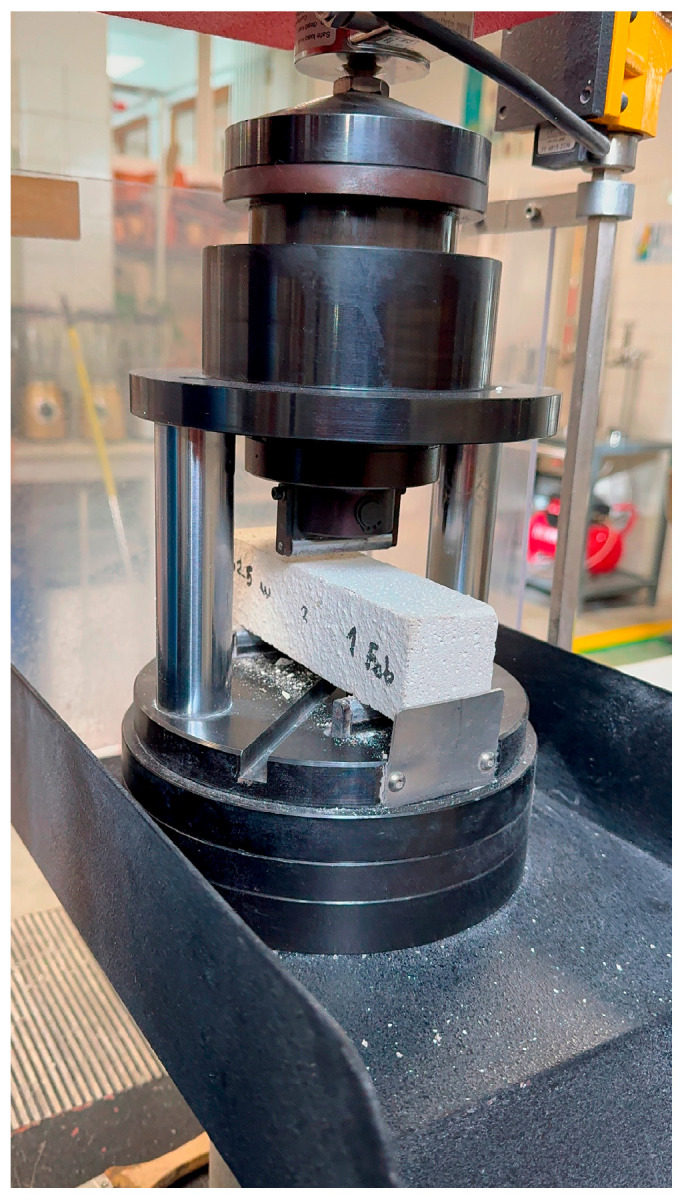
Flexural test.

Three flexural tests were performed for each water-cement ratio to ensure repeatability (Figure 2).

**Figure 3:**
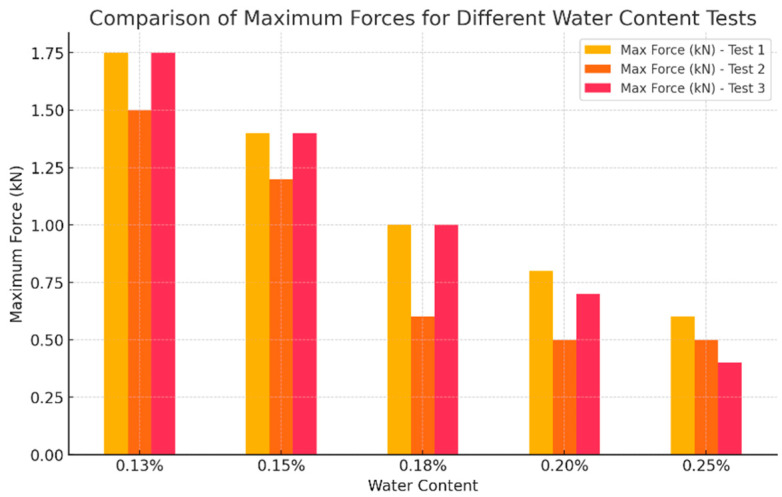
Comparison of Maximum Forces for Different Water Content Tests.

Figure 3 shows the comparison of maximum stresses for different water content tests (0.13 to 0.25).

Flexural tests clearly demonstrate that the water–cement ratio is a decisive parameter in the mechanical performance of mortar mixtures. Workability for 3D applications was evaluated through a combination of direct observation during extrusion trials and simple printing tests using elementary geometries (straight beads and stacked layers). A mix was considered suitable when it could be extruded continuously, retain its shape without collapse, and allow the deposition of successive layers without noticeable deformation. For the mixture with a *w*/*c* ratio of 0.18, stable extrusion, good cohesion, and the ability to stack up to five layers without shape loss were observed, justifying its selection as the optimal formulation. This ratio stands out as the most appropriate for structural applications, as it provides improved flexural strength while maintaining adequate workability for 3D printing.

### 2.3. Specimen Preparation

A water-to-cement ratio of 0.18 was used for the preparation of the specimens, as specified in the previous section.

#### 2.3.1. Mixing Procedure

(a) Dry Mixing: The contents of the CyBe mortar bag (a pre-blended mix of cement and sand) were first homogenized in a vertical-shaft mixer for approximately 1 min.
(b) Water Addition: Water was added gradually to achieve the target *w*/*c* ratio. The water supplied is potable and comes from the urban network of the city of Madrid (Spain). The flow rate is as specified in the material specifications: 300 L per hour at a temperature between 13 °C and 20 °C (55–68 °F).
(c) Homogenization: Mixing continued for an additional 2–3 min until a uniform, pumpable consistency was achieved. The temperature in the workspace was kept within the specified limits: between 5 °C and 30 °C (41–86 °F).

All specimens (both 3D-printed and cast) were cured in a controlled environment at 20 ± 2 °C with a relative humidity of ≥90%. Specimens remained in these conditions until testing at designated ages in 28 days to ensure uniform hydration and strength development [20].

#### 2.3.2. Manufacture of Specimens

The printers commonly used for additive manufacturing technologies in construction include gantry 3D printers, portal-type printers, and robotic arm printers. The most suitable for large-scale printing are portal-type printers, as they enable the construction of large-scale structures [118]. However, for this study, a robotic arm printer installed inside a building was chosen, as it allows for more precise control over temperature and humidity conditions and is particularly suited for producing specimens for subsequent testing.

**Figure 4:**
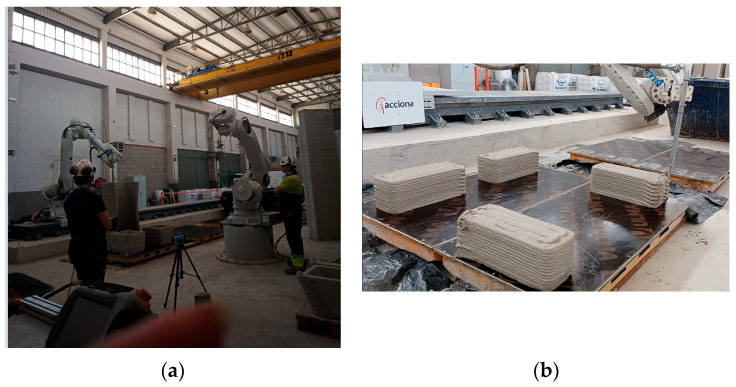
Manufacture of 3D printed test tubes in the Makerspace of Acciona’s Digital Innovation Hub: (**a**) Robotic arm; (**b**) Manufacture of specimens.

Both traditional and 3D printed specimens were manufactured at the Markerspace of Acciona’s Digital Innovation Hub (Torrejon de Ardoz, Spain) [119]. They were made with an ABB IRB 6740 robotic arm (ABB IRB 6740, ABB Robotics & Discrete Automation, Västerås, Sweden) combined with 3D printing technology [120] (Figure 4).

Each specimen was produced with a 20 cm × 20 cm cross-section and 50 cm length, following standard practice for structural testing. Traditional specimens were made by pouring mortar into molds, while 3D-printed units were created layer-by-layer using an extrusion-based additive manufacturing process. Both sets of specimens were cured under identical conditions for 28 days to ensure equal comparison and bending.

#### 2.3.3. Flexural Test

The flexural test was carried out to evaluate how traditionally molded and 3D-printed specimens respond under bending loads. It is performed by applying a three-point load to hardened mortar prisms until they fail.

The flexural tests were conducted at the Materials Laboratory of the School of Building Engineering at the Polytechnic University of Madrid.

To perform the test, a universal testing machine (UTM) applies a three-point bending configuration: two supports beneath the specimen and a load nose at the midpoint [52]. In this case, the machine used for the test is the Ibertest flexure testing machine, model TK100-500 (Ibertest S.A., Daganzo de Arriba, Madrid, Spain).

For the flexural tests, the relevant standards were reviewed. Since there are no specific standards for 3D-printed mortars [121], the following standards were considered in this research: ASTM C78/C78M [19] and UNE-EN 12390-5 [122].

First, specimen measurements are taken, particularly the cross-sectional dimensions required for the bending strength calculation, and the specimen is weighed on a scale for subsequent identification.

**Figure 5:**
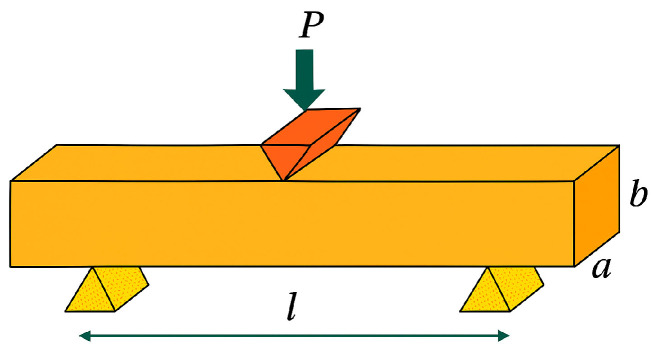
Positioning of the supports and application of the force in the center.

The loading device consists of two parallel supports for the specimen and a bending tip, which applies the load to the specimen at the center point between the supports (Figure 5).

**Figure 6:**
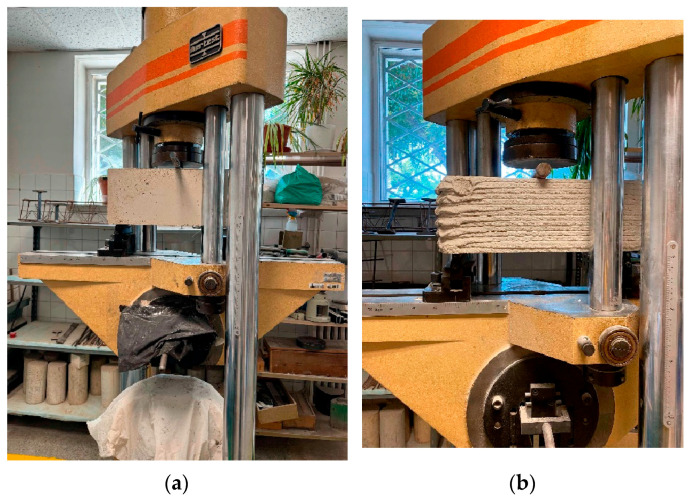
Application of force in the bending test: (**a**) Traditional way specimen; (**b**) 3D printed specimen.

According to the test requirements, both the supports and the bending tip must be fixed, rotating, or swiveling in order to carry out the test in accordance with the relevant specifications. The span between supports is 450 mm, and the roller diameter is 25 mm. The bearings are made of steel and have a cylindrical shape to minimize friction and allow free rotation of the specimen (Figure 6).

In order to minimize friction effects during the test, the supports may be mounted rotatable about their longitudinal axis. To ensure that the bending tip and the supports are parallel to the specimen, they can be tiltable.

The specimens under investigation are supported at two points and the load is applied at the center of the span and, therefore, the bending moment M_f_ is calculated as follows:

M_f_ = (P × l)/4 (1)

The resistant moment W for a square section specimen as the one used is:

W = (a × b^2^)/6 (2)

where a is the width of the specimen and “b” is the height of the specimen, both of which form the cross-sectional area of the specimen in which the breakage is to occur

Substituting into Equation (1) yields

σ_f_ = M_f_/W = (3 × P × l)/(2 × a × b^2^) (3)

If the load P is measured in N and the distances in mm, the bending strength σ_f_ is expressed in MPa.

**Figure 7:**
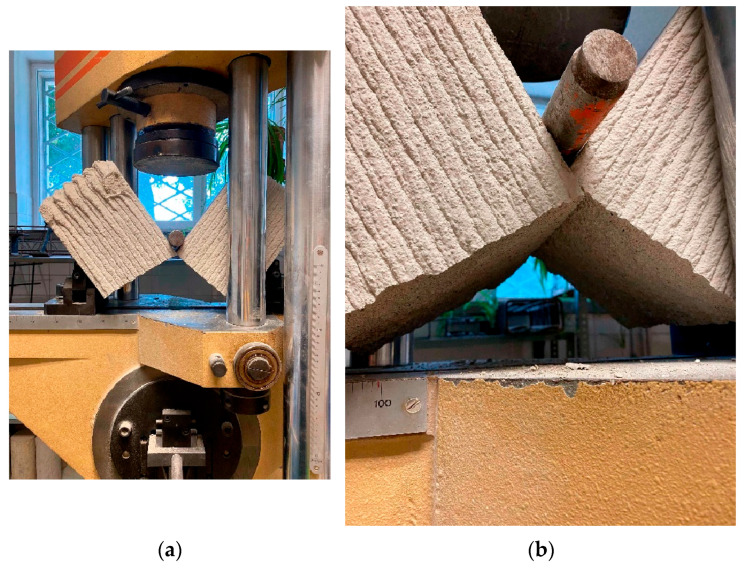
Flexural test of a 3D printed specimen: (**a**) Fracture of the specimen; (**b**) Detail of the flexural fracture.

The deformation that the part undergoes before reaching the breaking point is observed in the area where the force is being applied and in the lower supports (Figure 7).

The bending strength is calculated as the arithmetic mean of individual results, expressed, after rounding to the nearest 0.1 MPa.

## 3. Results

**Figure 8:**
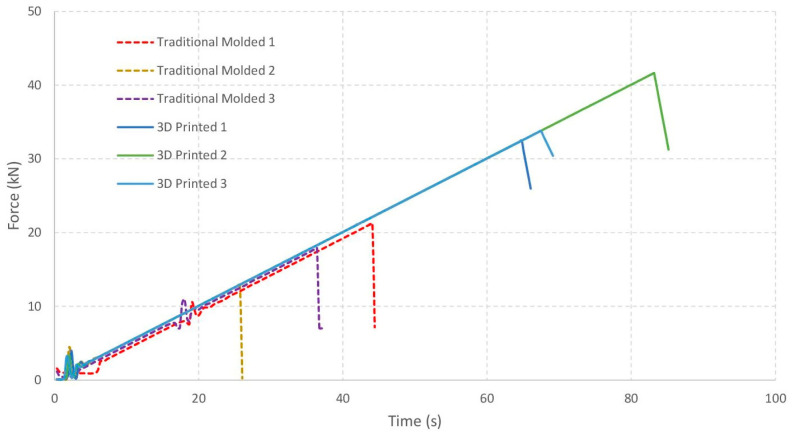
Force vs. time in the flexural test.

The following figure shows the force versus time graphs for the samples analyzed (Figure 8).

Below is the analysis of stress and strain for each unit based on the given dimensions (20 cm × 20 cm cross-sectional area and 50 cm length). The stress and strain are calculated using the peak force data from the experiments (Table 3).

The estimated strain was calculated from the central deflection using the formula for maximum flexural strain under a central load:

ε = (6·δ·h)/L^2^ (4)

where δ is the central deflection (mm), h is the height of the specimen’s cross-section (mm), and L is the span between supports (mm).

**Figure 9:**
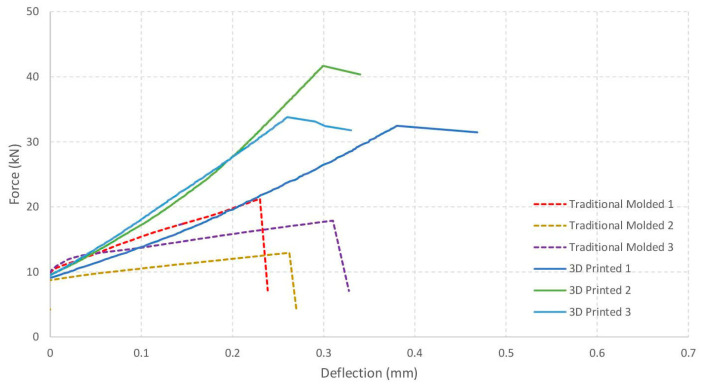
Force vs. deflection in the flexural test.

The following figure shows the force versus deflection graphs for the samples analyzed (Figure 9).

A table has been prepared showing fracture energy metrics derived from the area under the load-deflection curves, which provide a quantitative basis for comparing failure mechanisms (Table 4).

## 4. Discussion

The results of this study demonstrated that cast and 3D-printed specimens exhibit distinct behaviors under flexural loading. The 3D-printed specimens showed greater flexural strength than those manufactured using traditional methods. In the cast samples, the maximum stress ranged from 1.23 MPa to 1.78 MPa, indicating good strength of these materials. In contrast, although the 3D-printed samples exceeded the maximum stresses of the cast specimens, they exhibited a wider range of values, with a minimum maximum stress of 2.77 MPa and a maximum of 3.76 MPa.

The time required to reach the maximum load was also longer in the 3D-printed specimens, suggesting that these samples required a more extended loading path before failure or the onset of plastic deformation occurred. This behavior may be associated with a greater capacity to redistribute stresses prior to damage localization.

When examining the maximum strain behavior, clear differences were observed between the two groups. The cast samples exhibited strain values ranging from 1.37 × 10^−3^ to 1.84 × 10^−3^, reflecting a generally limited deformation capacity. In contrast, the 3D-printed specimens showed higher strain values, between 1.53 × 10^−3^ and 2.26 × 10^−3^, corresponding to an average increase of approximately 15–25% compared with the conventionally cast samples.

Furthermore, the results allow for an objective comparison of the failure mechanisms of cast and 3D-printed cement mortars. The load–deflection curves reveal clear differences in the response of the two types of specimens. In the cast samples, the total energy absorbed before failure ranged from 4.26 to 4.84 kN·mm, with post-peak energy values between 0.108 and 0.219 kN·mm, indicating a generally brittle response with limited energy dissipation after crack initiation. In contrast, the 3D-printed specimens exhibited total energy values between 7.79 and 10.41 kN·mm. This higher energy absorption, together with the significantly greater post-peak energy (1.70–2.82 kN·mm), reflects a more gradual loss of load-bearing capacity and suggests a mixed brittle–ductile failure mechanism. This behavior indicates that the 3D printing process may promote localized energy redistribution prior to complete failure.

In conclusion, the flexural test results confirm that the manufacturing process influences the mechanical behavior of the tested cement mortar specimens. The cast samples predominantly exhibited brittle fractures, a characteristic that may be advantageous in applications requiring predictability and stability under load. By contrast, the 3D-printed specimens displayed a combination of brittle and ductile behavior. An increase in relative deformation prior to failure was observed in the 3D-printed samples, indicating greater flexibility. This type of behavior may be beneficial in applications that require enhanced energy absorption capacity.

Overall, improving the quality of the printing process and employing techniques such as the incorporation of reinforcements or optimizing interlayer adhesion could enhance the performance of 3D-printed samples. In this context, the incorporation of expansive additives, such as environmentally friendly lithium slag, represents an effective strategy to improve deformation control, reduce crack formation, and increase mortar durability, especially under shrinkage conditions or post-peak loading [123].

As a future line of research, data fusion approaches could be explored to establish meaningful correlations between process signals and mechanical performance [124]. In the specific context of 3D-printed mortars, this would involve integrating process parameters (e.g., printing speed, temperature, humidity, curing time), structural characteristics (e.g., microstructure, porosity, layer orientation, density), and resulting properties (e.g., flexural strength, compressive strength, durability). Such integrative models may enhance predictive capabilities and support the optimization of printing strategies for improved material performance.

## 5. Conclusions

The findings of this study confirm that the mortar used in 3D printing is a viable and efficient alternative for structural fabrication, enabling layer-by-layer construction with high precision and adaptability. Comparing traditionally cast and 3D-printed specimens revealed that the manufacturing process significantly influences the mechanical response of cement-based materials.

Variations in the water-to-cement (*w*/*c*) ratio were found to significantly affect performance. Lower ratios (0.13–0.15) produced higher flexural strengths, while higher ratios (≥0.20) improved workability at the expense of mechanical capacity, highlighting the need to balance printability with mechanical requirements.

The findings of this study confirm that the manufacturing process has a decisive influence on the mechanical performance of cement mortar specimens. The 3D-printed samples exhibited higher maximum flexural stress values (2.77–3.76 MPa) than the traditionally molded ones (1.23–1.78 MPa), although with greater variability due to differences in interlayer bonding and print quality. In contrast, the molded specimens showed consistent strength and uniform behavior, reflecting good material homogeneity.

In terms of deformation, the molded specimens displayed a relatively limited strain capacity (1.37 × 10^−3^ to 1.84 × 10^−3^), whereas the 3D-printed samples reached higher strain values (1.53 × 10^−3^ to 2.26 × 10^−3^), indicating greater flexibility prior to failure. The load–deflection curves further revealed contrasting fracture mechanisms: molded specimens exhibited abrupt post-peak load drops and low energy dissipation (0.108–0.219 kN·mm), characteristic of brittle failure, while 3D-printed samples showed a more gradual post-peak response and higher post-peak energy values (1.702–2.824 kN·mm), indicative of a mixed brittle–ductile failure mode with enhanced energy absorption.

These results demonstrate that, when printing parameters are properly controlled, additive manufacturing can achieve equal or superior mechanical performance compared to conventional molding, particularly in terms of toughness and ductility. Molded specimens, due to their structural uniformity and predictable fracture behavior, remain suitable for standardized or load-critical applications. However, the increased deformation capacity and post-peak resistance of 3D-printed specimens make them advantageous for applications requiring energy absorption, damage tolerance, or impact resistance.

## Figures and Tables

**Table 1:** Material Properties.

Property		Standard
Color	Concrete grey	
Grain size	0–3 mm	DIN EN 933-1 [103]
Consumption	approx. 1.5 kg per 1.7 s–5 s (corresponds to a layer of 1 m length, 40 mm width and 20 mm height and depends on printing speed ranging 200–600 mm/s)	
Layer thickness	variable, standard 20 × 40 mm (h × w), depending on the selected nozzle, preliminary testing required	
Setting time	initial set approx. 3 min final set approx. 5 min	DIN EN 196-3 [104]
Load bearing	after 60 min	
Density (p)	Hardened approx. 2100–2200 kg/m^3^	DIN EN 12390-13 [105], DIN EN 12390-7 [106], DIN EN 12504-1 [107]
Thermal resistance (at mean temperature 35 °C)	0.054 (m^2^ °K)/W	ASTM C177:10 [108]
Thermal conductivity (λ) (At various temperatures, based on corresponding standard)	0.78 ^1^–0.979 ^2^ W/(m·K) ^1^ Performed on 3D printed specimen ^2^ Performed on densified lab specimen	DIN EN 12667 [109] ASTM C177:10
Specific heat capacity (c)	1.10 J/(g·K) ± 0.06	Determined with Macro-DSC
Thermal expansion coefficient (m)	15.0 × 10^−6^/K	DIN EN 1770 [110]
Depth of water penetration	23 mm	DIN EN 12390-8 [111]
pH value	12	
Sulfate resistance	Requirement fulfilled; High chemical resistance	According to W. Wittekindt [112]
Fire classification	Class A1: non-combustible	DIN EN 13501-1 [113]; acc.to EN ISO 1182 [114] and EN ISO 1716 [115]

**Table 2:** Oxide composition.

Component	Mass %
SiO_2_	22.7
Fe_2_O_3_	3.2
CaO	56.7
SO_3_	2.9
MgO	1.8
Na_2_O	0.98
K_2_O	0.71
Al_2_O_3_	8.2
Minor Oxid.	2.81

**Table 3:** Stress and Strain Analysis for Traditional and 3D-Printed Specimens: Peak Force, Time to Peak, Central Deflection at Peak Force, Estimated Stress, and Estimated Strain.

Specimen	Peak Force (kN)	Time at Peak Force (s)	Central Deflection at Peak Force (mm)	Estimated Stress (MPa)	Estimated Strain (-)
Traditional Molded 1	20.24	44.09	0.23	1.78	1.37 × 10^−3^
Traditional Molded 2	13.93	25.72	0.26	1.23	1.55 × 10^−3^
Traditional Molded 3	17.88	36.41	0.31	1.58	1.84 × 10^−3^
3D Printed 1	31.46	64.80	0.38	2.77	2.26 × 10^−3^
3D Printed 2	42.65	83.17	0.30	3.76	1.76 × 10^−3^
3D Printed 3	33.79	67.47	0.26	2.98	1.53 × 10^−3^

**Table 4:** Failure mechanisms for Traditional and 3D-Printed Specimens: Total Energy and Post-Peak Energy.

Specimen	Total Energy (kN·mm)	Post-Peak Energy (kN·mm)
Traditional Molded 1	4.328	0.122
Traditional Molded 2	4.259	0.108
Traditional Molded 3	4.837	0.219
3D Printed 1	10.411	2.824
3D Printed 2	8.648	1.702
3D Printed 3	7.786	2.295

## Data Availability

The original data presented in the study are openly available in [e-cienciaDatos] at [https://doi.org/10.21950/P9JJ2P].
